# The outcome and cost of a capacity‐building training programme on the early recognition and referral of childhood cancer for healthcare workers in North‐West Cameroon

**DOI:** 10.1002/nop2.598

**Published:** 2020-08-26

**Authors:** Glenn Mbah Afungchwi, Peter Bernard Hesseling, Francine Kouya, Sam A. Enow, Mariana Kruger

**Affiliations:** ^1^ Mbingo Baptist Hospital Mbingo Cameroon; ^2^ Department of Paediatrics and Child Health Faculty of Medicine and Health Sciences Stellenbosch University Cape Town South Africa; ^3^ Regional delegation of Public Health Bafoussam Cameroon; ^4^ School for State Registered Nurses Bafoussam Cameroon

**Keywords:** Cameroon, capacity building, childhood cancer, early diagnosis, my child matters

## Abstract

**Aim:**

Early cancer diagnosis is necessary to improve survival rates. The aim of this study was to assess the outcome and cost of the childhood cancer training programme amongst healthcare workers.

**Design:**

This was a prospective pre–post study design, using questionnaires for pre‐ and post‐training testing. The warning signs of childhood cancer were used as the main teaching content to improve recognition and early diagnosis.

**Methods:**

Pre‐training and post‐training knowledge, as well as attitude questionnaires, was administered at the beginning and at the end of each training workshop. Paired samples *t* test and chi‐square were used to compare the change in knowledge and differences between groups.

**Results:**

The overall percentage knowledge score increased from 51%–85% (*p* < .001). The doctors had a better knowledge score than the nurses in the pre‐test (70% versus 50%, *p* = .008), but there was no significant difference in the post‐test scores. The cost of training was €25.06 per healthcare worker.

**Conclusion:**

We recommend similar training programmes in public health to improve early diagnosis of childhood cancer.

## 1 BACKGROUND

There are vast disparities in childhood cancer survival rates between developed countries and low‐ and middle‐income countries (LMIC). Great progress has been made over the past half‐century in the care of children with cancer, with 80% of children with cancer cured in high‐income countries (HIC). However, the survival rates in developing countries still range between 20%–70% (Chantada, Lam, & Howard, 2019). This makes the plight of children with cancer a global concern, as 80% of children diagnosed annually with cancer live in LMIC (Israëls et al., 2013).

To improve childhood cancer survival in LMIC, paediatric oncology experts have suggested actions that include the promotion of early diagnosis; capacity‐building training programmes, effective childhood cancer care programmes; a reduction in treatment‐related deaths; and a reduction in treatment abandonment (Friedrich et al., 2015; Mostert et al., 2011). Twinning partnerships between HIC and LMIC have led to a significant improvement in local childhood cancer management capacity, drug availability and the development of locally adapted protocols [5–8].

The Stellenbosch University‐Cameroon Baptist Convention Health Services (CBCHS) twinning programme has operated a childhood cancer treatment programme since 2003, with two treatment centres in the north‐west region. Both centres provide free treatment and support for the feeding and transportation of all children younger than 15 diagnosed with cancer. Hesseling et al. reported that up to 84% of these patients presented at the hospital with advanced‐stage disease and only 16% with limited disease. The event‐free survival was 100% for stage I, 85% for stage II, 60% for stage III and 27% for stage IV disease (Hesseling et al., 2012). Early diagnosis with more limited disease therefore implies a better chance to be cured with the currently available facilities and expertise (Miller et al., 2019).

Delays in the health system have been shown to be a major contributor to delays in the diagnosis of childhood cancer [11–13]. A previous survey of 50 childhood cancer patients at Mbingo Baptist Hospital in Cameroon showed that 70% of them presented to the treatment centre more than three weeks after the onset of disease, with the delay being shorter for patients referred by health professionals than for self‐referred patients (Kouya, 2012). Brown et al. reported that the delay in diagnosis was longer when childhood cancer patients in Nigeria visited several health facilities (Brown, Adeleye, & Ibeh, 2015).

With a low physician to population ratio in Cameroon of 0.08 per 1,000 of the population (Central Intelligence Agency, 2018), most of the health care in rural settings is left in the hands of nurses and community healthcare workers (National Institute of Statistics, 2012). Traditional medicine is also a relatively popular choice for health care in Cameroon and therefore recognized by law and integrated into the healthcare system (Asonganyi, 2013). Fifty‐five per cent of children with Burkitt lymphoma in North‐West Cameroon primarily visit traditional healers as the first choice for care [18]. The traditional healers may make incorrect diagnoses and apply various forms of treatment that are ineffective and often painful and/or harmful to children (Afungchwi, Hesseling, & Ladas, 2017). This situation highlights the critical importance of empowering rural nurses and community healthcare workers to be frontline actors to educate communities, as well as to identify children with cancer for early referral and treatment (Mbah Afungchwi & Challinor, 2016).

An important tool is to train healthcare workers, especially nurses and community healthcare workers, to recognize the early warning signs and symptoms of childhood cancer. It is also important to improve the linkage between community healthcare workers and cancer referral centres and to improve transport support for children with cancer (Buckle et al., 2013). A training programme in rural South Africa has led to an increase in referrals and average annual diagnoses, although not necessarily decreasing the delay in diagnosis (Poyiadjis, Wainwright, Naidu, Mackinnon, & Poole, 2011). The effect on patient outcome has not been measured. Similarly, educating healthcare professionals in Botswana has led to improvement in healthcare workers’ knowledge of childhood cancers, with concomitant improved referral rates (Slone, Ishigami, & Mehta, 2016a).

This study aimed to assess the outcome and cost of a SANOFI‐sponsored training programme with the title, "My Child Matters," regarding knowledge of childhood cancer amongst healthcare workers in the north‐west region of Cameroon.

## 2 METHODS

This was a prospective pre–post study design. The Sanofi Espoir "My Child Matters" programme (Sanofi Espoir Foundation, 2020) supported the establishment of a network of 133 healthcare providers (including 20 traditional healers) trained to potentially identify childhood cancer cases in six health districts of North‐West Cameroon in 2016. Head nurses and medical doctors of all the leading health centres and three prominent traditional healers from each health district were invited to participate in the training workshops at their various district health services’ headquarters. The selection of participating centres was based on the recommendation of the district medical officers and district supervisors. The healthcare facilities included healthcare centres headed by nurses, sub‐divisional medical centres headed by physicians and a district hospital headed by physicians. Participants included head nurses of all lead health centres, medical officers of all sub‐divisional medical centres and district hospitals were invited to participate in the training, three prominent traditional healers from each health district and the district supervisory teams. Of this group of participants, 18 were selected as "ambassadors" and equipped with mobile phones and mobile money accounts to communicate with childhood cancer centres and to refer patients promptly for diagnosis and treatment.

**Figure 1:**
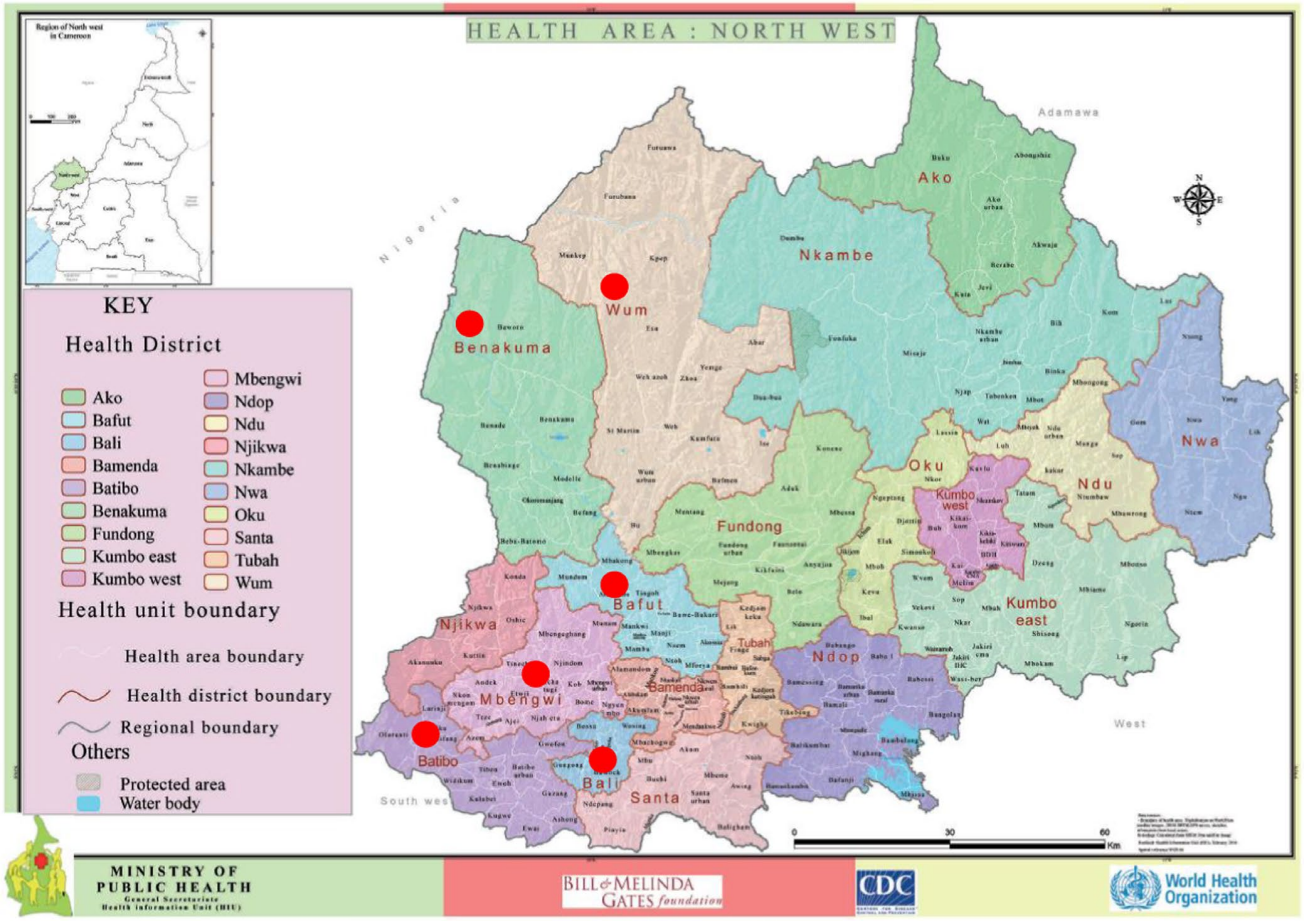
Map of North‐West Cameroon, showing health districts indicated by red dots (Bafut, Mbengwi, Batibo, Benakuma, Wum, Bali). Source: Ministry of Public Health, Cameroon. https://www.dhis‐minsante‐cm.org/portal/

The training programmes were held between June and September 2016 and covered six health districts, namely Mbengwi, Bafut, Batibo, Bali, Wum and Benakuma, in North‐West Cameroon, with a total surface area of 6,601 km^2^ and a total population of 439,956 (Northwest regional delegation of public health, 2015) (Figure 1). The workshops lasted 4 hr and consisted of PowerPoint presentations with case studies and pictures, followed by group discussions on effective referrals. The topics covered included an explanation of what cancer is, the common childhood cancers and their presentations and a discussion on how to effectively refer a child with cancer to a treatment centre. Participants were provided with brochures and flyers on the warning signs of childhood cancer that contained the Saint Siluan warning signs of cancer in children, developed by the South African Children's Cancer Study Group (Poyiadjis et al., 2011), with pictures of example cases. The facilitators of the training workshops were paediatric oncology nurses and nurse practitioners from two childhood cancer centres in the north‐west region. The project design and workshop content development were overseen by the oncologist at Mbingo Baptist Hospital and the paediatric oncologist from Stellenbosch University.

A pre‐training knowledge and attitude questionnaire (Appendix 1) was administered to the attending healthcare professionals at the beginning of each training workshop and again at the end of the training workshop in the same group (Appendix 2), to assess initial knowledge and post‐training knowledge. The questionnaire included questions on the education and experience of the healthcare worker; the types of childhood cancers; signs of childhood cancer; and childhood cancer treatment sites in Cameroon. It also included attitude‐related questions: whether they had ever referred a child with cancer and whether or not they educated their communities about childhood cancer. The Saint Siluan warning signs for childhood cancer was used to grade the correctness of their knowledge of childhood cancer warning signs (Poyiadjis et al., 2011). Participation in the pre‐ and post‐test was optional and on provision of informed consent. The knowledge of risk factors of cancer was graded based on published literature on cancer aetiology, while knowledge on treatment sites and types of childhood cancers were graded based on expert knowledge. The cost of the entire capacity‐building training programme was recorded on a spreadsheet.

### 2.1 Statistical analysis

Descriptive statistics were used to present the characteristics of the study participants. The five questions of the questionnaire which assessed knowledge had a moderate internal consistency, with a Cronbach's alpha coefficient of 0.6. The IBM Statistical Package for the Social Sciences (SPSS) version 25 was used to analyse the data. The paired samples *t* test was used to compare the change in knowledge scores before and after the training. A chi‐squared test was used to compare the difference in scores between physicians and nurses. The level of statistical significance was set at p values less than 0.05. Atlas.ti version 8 was used to code and group free‐text responses into themes. The cost was analysed for various aspects of the project, and the total cost per participant trained was estimated.

### 2.2 Ethics

Ethics clearance was obtained from the Cameroon Baptist Convention Health Services Institutional Review Board (IRB2018‐42) and the Health Research Ethics Committee of Stellenbosch University (project ID 8,041, HREC reference number S18/08/163). Responding to the questionnaire was completely voluntary, following the reading of the participant information sheet and consent to participate.

## 3 RESULTS

### 3.1 Characteristics of participants

A total of 113 healthcare professionals and 20 traditional healers were trained across the six districts. Traditional healers were left out of the pre‐ and post‐training tests because most of them were not literate and could not complete the paper‐based questionnaires. Twenty eligible respondents (17.7%) did not participate. Ninety‐three of the participants (82.3%), all healthcare workers, completed both the pre‐training and post‐training test. Most were from primary healthcare centres (78.5%), followed by district hospitals (17.2%) and sub‐divisional medical centres (4.3%). Most were State Registered Nurses (three years’ training; 40%), followed by nursing assistants (ten months’ training; 20%), brevete nurses (two years’ training; 15.6%), bachelor's degree nurses (four years’ training; 10%), nurse aides (7.4%) and physicians (6.7%). Most participants had either between five and 10 years’ (35.5%) or more than 10 years’ (34.4%) working experience in health care, while 17.2% had worked between two and five years and 12.9% for less than two years. Ninety‐six of the participants (85%) consulted children in their day‐to‐day practice. Nine thousand brochures were distributed amongst the trainees, as were printed handouts of all the modules taught.

Eighteen per cent of the respondents in the pre‐training test did not have any knowledge of childhood cancers. Those who had some knowledge of childhood cancers reported having acquired this knowledge during their training at their nursing or medical school (61%), from medical or nursing textbooks and journals (23.4%), from the Internet (22.3%), or from another physician or nurse (16%). A few (15.1%) had been giving health talks regarding childhood cancers in their communities (Table 1).

**Table 1:** Characteristics of participants

Participant characteristic	Frequency (%)
Type of facility where participant works (*n* = 93)	
Health centre	73 (78.5)
Sub‐divisional medical centre	4 (4.3)
District hospital	16 (17.2)
Level of training of participant (*n* = 90)	
Nurse aide	7 (7.4)
Nursing assistant	18 (20.0)
Brevete nurse	14 (15.6)
State Registered Nurse	36 (40.0)
BSc nurse	9 (10.0)
Physician	6 (6.7)
Participant's years of experience in health care (*n* = 93)	
0–2 years	12 (12.9)
2–5 years	16 (17.2)
5–10 years	33 (35.5)
More than 10 years	32 (34.4)
Source of childhood cancer knowledge	
Nursing or medical school	58 (61)
Another physician/nurse	15 (16)
Internet	21 (22.3)
Medical or nursing textbooks/journals	22 (23.4)
No knowledge	17 (18.1)
Participants who consult children (*n* = 90)	86 (95.6)
Participants who have been giving health talks on childhood cancers in their communities prior to training	14 (15.1)

### 3.2 Change in knowledge

The first part of the knowledge test included information of cancer‐causing physical agents such as radiation and sunlight; chemical agents such as tobacco smoke and pesticides; viruses and bacteria; canned foods and grilled goods; and genetic factors. The mean recognition score for these risk factors increased significantly, from 3.19 in the pre‐test to 4.21 in the post‐test (*p* < .001). Participants were asked to list four types of childhood cancers, and the mean score of correct answers for this question increased significantly, from 1.38 in the pre‐test to 2.94 in the post‐test (*p* < .001). In the pre‐test, 10 correct responses for cancer type (exact cancer names were not required) and their frequencies were identified: blood cancer (*N* = 51, 54.8%), bone cancer (*N* = 24, 25.8%), brain cancer (*N* = 16, 17.2%), Burkitt lymphoma (*N* = 11, 11.8%), eye cancer (*N* = 9, 9.7%), Kaposi sarcoma (*N* = 3, 3.2%), lymphoma (*N* = 10, 10.8%), nephroblastoma (*N* = 3, 3.2%) and neuroblastoma (*N* = 1, 1.1%). In the post‐test, the specific types of cancers were identified, with their frequencies as follows: retinoblastoma (*N* = 49, 52.7%), Burkitt lymphoma (*N* = 48, 51.6%), nephroblastoma/Wilms’ tumour (*N* = 35, 37.6%), osteosarcoma (*N* = 33, 35.5%), leukaemia (*N* = 31, 33.3%), Kaposi sarcoma (*N* = 21, 22.6%), lymphoma (*N* = 20, 21.5%), neuroblastoma (*N* = 2, 2.2%), Hodgkin's lymphoma (*N* = 1, 1.1%) and non‐Hodgkin's lymphoma (*N* = 1, 1.1%). Far fewer responded with the broad categories, such as blood cancer (*N* = 4, 4.3%), bone cancer (*N* = 11, 11.8%), eye cancer (*N* = 10, 10.8%) and brain cancer (*N* = 1, 1.1%),

The third section tested knowledge of the warning signs of childhood cancer (Table 2). The mean score for this question changed from 1.87 in the pre‐test to 3.60 in the post‐test on a point scale of 4 (*p* < .001). Finally, participants were asked to list dedicated paediatric oncology units in Cameroon. The mean score for this question increased from 1.37–2.91 on a scale of 3 (*p* = .087). Four correct sites were identified in the pre‐test, with the following frequencies: Mbingo Baptist Hospital (*N* = 49, 52.6%), Banso Baptist Hospital (*N* = 41, 44.1%), Chantal Biya Foundation Mother and Child Centre in Yaounde (*N* = 14) and Baptist Hospital Mutengene (*N* = 7, 7.5%). In the post‐test, the same four sites were listed in different frequencies, as follows: Mbingo Baptist Hospital (*N* = 84, 90.3%), Banso Baptist Hospital (*N* = 78, 83.9%), Chantal Biya Foundation Mother and Child Centre in Yaounde (*N* = 55, 59.1%) and Baptist Hospital Mutengene (*N* = 52, 55.9%). When asked if they thought it was possible to cure a child with cancer in Cameroon, 86% said yes in the pre‐test and 98% agreed in the post‐test (*p* = .019).

**Table 2:** Change in knowledge

Knowledge	Pre‐test	Post‐test	*p* value	*t*	*df*
Recognition of factors that predispose to cancer	*n* (%)	*n* (%)			
Physical agents like radiation and sunlight	68 (72.3)	89 (94.7)	<.001	4.635	93
Chemical agents like tobacco smoke and pesticide	81 (86.2)	93 (98.9)	.001	3.380	93
Viruses and bacteria	48 (51.1)	51 (54.3)	.534	0.624	93
Canned food/grilled foods	40 (42.6)	81 (86.2)	<.001	8.137	93
Genetic factors	63 (67)	82 (87.2)	<.001	4.318	93
Mean score of knowledge about cancer risk factors (max. 5)	3.19	4.21	<.001	8.071	93
Mean score of knowledge about types of childhood cancers (max. 4)	1.38	2.94	<.001	10.557	93
Mean score of knowledge about signs of childhood cancers (max. 4)	1.87	3.60	<.001	12.629	93
Mean score of knowledge about childhood cancer treatment centres in Cameroon (max. 3)	1.37	2.91	.087	1.730	93
Percentage overall score on knowledge items	50.88	84.79	<.001	17.747	93

When knowledge scores were compared between doctors and nurses in the pre‐test, there were no significant differences in the mean scores for knowledge regarding factors predisposing to cancer (*p* = .799), types of childhood cancers (0.137) or dedicated paediatric oncology units in Cameroon (*p* = .086). However, the doctors scored higher than the nurses regarding knowledge of the warning signs of childhood cancer [2.50 (*SD* 1.643) versus 1.31 (*SD*: 0.891), *p* = .137] and in the overall knowledge score [69.61 (*SD *= 29.159) versus 49.79 (*SD *= 16.218), *p* = .008]. In the post‐test, there was no significant difference in scores for types of childhood cancers (*p* = .438), signs of childhood cancers (*p* = .688), dedicated paediatric oncology units in Cameroon (*p* = .517) and overall knowledge score (*p* = .845). However, the nurses scored significantly higher (4.30, *SD *= 0.915) in identifying the risk factors for cancer than the doctors (3.50, *SD* 1.225), *p* = .047.

At the end of the training, 99% of the participants confirmed that they would be able to effectively refer childhood cancer patients to treatment centres. All (100%) participants thought that the training would benefit their community and their clinical practice, that they would teach their colleagues about childhood cancers and would be giving health talks on childhood cancer to their community members.

The length and form of higher education in terms of healthcare training were associated with improved childhood cancer knowledge, as there was a significant correlation between the participants’ form of training in health care and their mean score for knowledge about childhood cancer types, signs of childhood cancer and the availability of treatment all together (*p* = .005). However, these scores did not show a significant correlation with years of experience in health care (*p* = .692).

### 3.3 Costs

**Figure 2:**
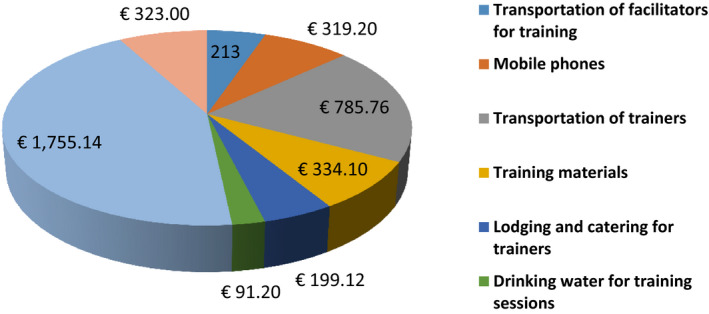
Cost distribution

As training programmes need financial support, the total costs were also documented. The total cost for the project was €4,476.32. The cost of training was €3,834.124, of which €518.32 was used to provide mobile phones and mobile money accounts for ambassadors to communicate with paediatric oncology unit teams and transport suspected cases, with an average of €25.06 per healthcare worker attending. The major part (48%) of the training cost was for the transportation of participants and catering, followed by 21% for the transportation of facilitators, 12% for printing brochures and leaflets, 9% for training materials, and 5% for lodging and catering for facilitators (Figure 2).

The participants provided several suggestions for improving the early diagnosis of childhood cancers in the region. The most common suggestions were awareness creation in communities (*N* = 19), quick referral of suspected cases to treatment centres (*N* = 18), training more healthcare providers (*N* = 12) and refresher training courses on the early diagnosis of childhood cancers (*N* = 10). Some participants suggested proper physical examination of children at consultation (*N* = 5), the designation of more focal persons for referrals (*N* = 4), the provision of more leaflets on warning signs to healthcare centres and communities (*N* = 3), the inclusion of childhood cancer as a disease of surveillance (*N* = 3) and improving collaboration with traditional healers (*N* = 3). A few suggested campaigns to actively search for children with signs of cancer in communities (*N* = 2), screening for lumps at Infant Welfare Clinics (*N* = 2), counter‐referral of referred patients after diagnosis and treatment (*N* = 2) and advocacy with community leaders and politicians (*N* = 2).

## 4 DISCUSSION

Capacity building is defined as any activity or modification aimed at or resulting in an improved outcome within a health system (Baillie, Bjarnholt, Gruber, & Hughes, 2009). Capacity building is used as a means to promote surveillance for diseases, notably communicable diseases in Africa, and has been shown to be effective in the surveillance of disease outbreaks (Durrheim, Harris, Speare, & Billinghurst, 2001). In Cameroon, capacity building within the Expanded Program on Immunization is used to strengthen the surveillance of diseases with epidemic potential, including poliomyelitis, yellow fever, measles and neonatal tetanus.

Cameroon has a three‐tier health system, the most basic part of which is the peripheral level, consisting of health centres, sub‐divisional medical centres and district hospitals (http://www.statistics‐cameroon.org/downloads/pets/2/Rapport_principal_Sante_anglais.pdf). It was the district level that was targeted for this capacity‐building project. The health professionals at this level have few specialized care services and therefore are usually abreast of most communicable diseases that require prompt management and are included in their minimum package of activity. Only 6.7% of our participants were physicians. This is not unexpected, given a doctor‐to‐population ratio of 0.08 per 1,000 (Central Intelligence Agency, 2018) compared with a nurse‐to‐population ratio of 0.8 per thousand (Global Health Workforce Alliance, 2020lliance, 2020) and the fact that, as reported in 2013 (World Bank, 2013), 40% of the country's doctors practice in the central region, where the capital city Yaounde is located. Most health professionals who attended the training were nurses, similar to the case in Botswana, although the ratio of nurse to physician in that setting was much smaller (1.6:1) than in this report (14:1) (Slone et al., 2016b). This highlights the importance of including nurses in capacity‐building training programmes in Africa to promote the early identification and prompt referral of childhood cancer patients. Noteworthy is the fact that 95.6% of the healthcare workers consulted children, irrespective of their level of healthcare training.

The Saint Siluan signs have also been used in South Africa (Poyiadjis et al., 2011) and in Botswana (Slone et al., 2016a) for educating healthcare providers on childhood cancer. Providing nurses with educational material has been shown to be effective in enhancing knowledge acquired from special training (David & Banerjee, 2010). In a group of medical students in South Africa, similar signs and symptoms were identified, with the most common ones being easy bruising, fatigue, unexplained bleeding, unexplained fever, weight loss and loss of appetite. Signs in the eye, like a white spot in the eye and a bulging eyeball, were also identified, which was not the case amongst our participants in the pre‐test (Geel et al., 2017).

Training programmes regarding the warning signs of childhood cancer are effective tools to increase awareness and referral of patients to healthcare centres in LMICs. Following similar training on early warning signs in South Africa, the number of patients diagnosed in the region increased for both solid and haematologic malignancies (Poyiadjis et al., 2011). In Honduras, a nationwide retinoblastoma campaign was successful in reducing diagnostic delays (Leander et al., 2007).

The Botswana training programme reported an average cost of $11.20 per participant (Slone et al., 2016a), while the cost for this project in Cameroon was €25.06 ($ 27.64) per healthcare provider. The biggest burden of the cost was transportation, lodging and catering for trainees and trainers. Distances between health centres in the districts are long, and only 7% of the country's roads were paved in 2016 (Central Intelligence Agency, 2018). Although each training workshop was done in about four hours, it required a whole day for the trainees and an overnight stay for the trainers. This mirrors the difficulties experienced by patients who need to travel to hospitals out of their home districts and stay for several months to receive treatment.

The contribution of community participation is essential for disease surveillance (Ndiaye, Quick, Sanda, & Niandou, 2003). In Cameroon, the use of trained community‐directed distributors (CDDs) in onchocerciasis control has proven to be reliable and cost‐efficient (Tanya et al., 2012). If the trained health professionals transfer childhood cancer knowledge to community members, it is expected that the delay from onset of disease to diagnosis will also significantly reduce late diagnosis, with the potential to improve cure rates.

### 4.1 Limitations

It is difficult for the clinical team to conduct frequent outreach training while maintaining the quality of clinical care at the centres due to limited human resource capacity (Slone et al., 2016a). The trainers in this project were all nurses and nurse practitioners, with no paediatric oncologist physically available to train in the districts. Secondly, the training programme was intensive, with teaching of childhood cancer signs, types and referrals within four hours with the potential risk that the knowledge would not be retained, necessitating regular refresher courses. Given that the traditional healers could play a significant role in early diagnosis of childhood cancer, the approach of this study with self‐administered questionnaires failed to assess the outcome of the training amongst traditional healers who participated in them. Future similar initiatives should consider an in‐person assessment component to capture the outcome for traditional healers. Finally, the study only measures immediate outcome of the project and does not re‐evaluate trainees to see how much of the acquired knowledge is retained over time.

## 5 CONCLUSION

Delays in diagnosis can be either due to patient delay or healthcare system delay. In Nicaragua (De Angelis et al., 2012) and South Africa (Stefan & Siemonsma, 2011), healthcare system delay was shown to be a major problem, hence the need to implement educational initiatives to improve childhood cancer knowledge amongst first‐line healthcare providers (Rodriguez‐Galindo, Friedrich, Morrissey, & Frazier, 2013). Our capacity‐building project significantly improved childhood cancer knowledge amongst healthcare workers in the six districts covered. These health professionals are most often managing children with infectious diseases and malnutrition (WHO, 2015). Teaching them about childhood cancer makes them realize the reality of cancer in children in Cameroon and increases the likelihood of early diagnosis of childhood cancer and potential improved cure rates. It thus increases their suspicion index for childhood malignancies and gives them clear knowledge of how to facilitate access to prompt diagnosis and life‐saving treatment for these children. Joko‐Fru et al. (Joko‐Fru et al., 2018) assert that survival in the case of common and curable cancers in Africa is poor and could be improved by increased support for activities around cancer detection, treatment and registration.

Our recommendation is for complete integration of training programmes into the public health system, with incorporation into the district health service activities involving dialogue structures in this system. To do this effectively, it will be necessary to train trainers at the district level to serve as focal persons for childhood cancer. These trainers could then be empowered to conduct routine refresher training with healthcare providers in their various districts. A clear monitoring and evaluation framework for childhood cancer suspicion and referral should be developed together with the district medical officers, which fits well within the current monitoring and evaluation procedures in the region. Cancer registries should also be developed and used to track trends and delays in childhood cancer diagnosis in the region.

## CONFLICT INTERESTS

There is no conflict of interest.

## Appendix 1

### Pre‐training questionnaire for capacity‐building training project

#### Improving referral rates for early diagnosis of childhood cancers in north‐west Cameroon by use of trained ambassadors and mobile money transfer

##### Pre‐training test

Please provide a code number (code name) at the bottom of the page.

1. What type of facility do you work in?
  - Health centre
  - Subdivisional medical centre
  - District hospital
  - Regional hospital
2. What is your level of training in health care
  - Nurse Aide
  - Nursing assistant
  - Brevete Nurse
  - State registered nurse
  - BNS nurse
  - Physician
3. How long have you been working in health care?
  - 0–2 years
  - 2–5 years
  - 5–10 years
  - More than 10 years
4. Do you ever consult children?
  - Yes
  - No
5. What are some common causes of cancer (Tick all correct answers)
  - Physical agents like radiation and sunlight
  - Chemicals like tobacco smoke and herbicides
  - Canned food/grilled foods
  - Some viruses, bacteria and parasites
  - Genetic factors
6. List four cancers that occur in Children _____________________________________________________________________________________________________________________
7. State four signs that are common in childhood cancers __________________________________________________________________________________________________________________________
8. List three centres in Cameroon where childhood cancer is managed.__________________________________________________________________________________________________________________
9. Can childhood cancers be cured in Cameroon?
  - Yes
  - No
10. Where did you get your knowledge on childhood cancers?(Please tick all that apply)
  - From Nursing/medical school
  - From another nurse/ physician
  - From the media
  - From the Internet
  - From text books/journals
  - I have no knowledge about childhood cancers
11. Do you ever give health talks about cancers in children in your community?
  - Yes
  - No
12. Would you like to have more knowledge about childhood cancer?
  - Yes
  - No

## Appendix 2

### Post‐training questionnaire for capacity‐building project

#### Improving referral rates for early diagnosis of childhood cancers in north‐west Cameroon by use of trained ambassadors and mobile money transfer.

##### Post‐training test

Please use the same code number (code name) from your pre‐training test.

1. What are some common causes of cancer (tick all correct answers)
2. Physical agents like radiation and sunlight
3. Chemicals like tobacco smoke and herbicides
4. Canned food/grilled foods
5. Some viruses, bacteria and parasites
6. Genetic factors
7. List four cancers that occur in Children
  _______________________________________________________________________________________________________
8. State four signs that are common in childhood cancers
  ________________________________________________________________________________________________________
9. List three centres in Cameroon where childhood cancer is managed.
  ________________________________________________________________________________________________
10. Can childhood cancers be cured in Cameroon?
  - Yes
  - No
11. Did this training provide you with knowledge that could benefit your community and your practice?
  - Yes
  - No
12. Will you be giving health talks about childhood cancers in your community in future?
  - Yes
  - No
13. Can you effectively refer childhood cancer patient through local ambassadors?
  - Yes
  - No
14. Will you be teaching colleagues how to identify children with cancer in future?
  - Yes
  - No

Please write down any suggestions you have for improving early diagnosis of childhood cancer
